# Mono, bi- and tri-exponential diffusion MRI modelling for renal solid masses and comparison with histopathological findings

**DOI:** 10.1186/s40644-018-0178-0

**Published:** 2018-11-26

**Authors:** Sophie van Baalen, Martijn Froeling, Marino Asselman, Caroline Klazen, Claire Jeltes, Lotte van Dijk, Bart Vroling, Pieter Dik, Bennie ten Haken

**Affiliations:** 1 Magnetic Detection & Imaging, University of Twente, Drienerlolaan 5, 7522 NB Enschede, Netherlands; 2 Radiology, University Medical Center Utrecht, Heidelberglaan 100, 3584 CX Utrecht, Netherlands; 3 Urology, Medisch Spectrum Twente, Koningsplein 1, 7512 KZ Enschede, Netherlands; 4 Radiology, Medisch Spectrum Twente, Koningsplein 1, 7512 KZ Enschede, Netherlands; 5 Pediatric Urology, Wilhemina Children’s Hospital, Lundlaan 6, 3584 EA Utrecht, Netherlands

**Keywords:** Magnetic resonance imaging, Diffusion magnetic resonance imaging, Diffusion tensor imaging, Kidney neoplasms

## Abstract

**Purpose:**

To compare diffusion tensor imaging (DTI), intravoxel incoherent motion (IVIM), and tri-exponential models of the diffusion magnetic resonance imaging (MRI) signal for the characterization of renal lesions in relationship to histopathological findings.

**Methods:**

Sixteen patients planned to undergo nephrectomy for kidney tumour were scanned before surgery at 3 T magnetic resonance imaging (MRI), with *T*_*2*_-weighted imaging, DTI and diffusion weighted imaging (DWI) using ten *b*-values. DTI parameters (mean diffusivity [MD] and fractional anisotropy [FA]) were obtained by iterative weighted linear least squared fitting of the DTI data and bi-, and tri-exponential fit parameters (*D*_*bi*_*, f*_*star,*_*and D*_*tri*_*, f*_*fast,*_*f*_*interm*_) using a nonlinear fit of the multiple b-value DWI data. Average parameters were calculated for regions of interest, selecting the lesions and healthy kidney tissue. Tumour type and specificities were determined after surgery by histological examination. Mean parameter values of healthy tissue and solid lesions were compared using a Wilcoxon-signed ranked test and MANOVA.

**Results:**

Thirteen solid lesions (nine clear cell carcinomas, two papillary renal cell carcinoma, one haemangioma and one oncocytoma) and four cysts were included. The mean MD of solid lesions are significantly (*p* < 0.05) lower than healthy cortex and medulla, (1.94 ± 0.32*10^− 3^ mm^2^/s versus 2.16 ± 0.12*10^− 3^ mm^2^/s and 2.21 ± 0.14*10^− 3^ mm^2^/s, respectively) whereas *f*_*fast*_ is significantly higher (7.30 ± 3.29% versus 4.14 ± 1.92% and 4.57 ± 1.74%) and *f*_*interm*_ is significantly lower (18.7 ± 5.02% versus 28.8 ± 5.09% and 26.4 ± 6.65%). Diffusion coefficients were high (≥2.0*10^− 3^ mm^2^/s for MD, 1.90*10^− 3^ mm^2^/s for *D*_*bi*_ and 1.6*10^− 3^ mm^2^/s for *D*_*tri*_) in cc-RCCs with cystic structures and/or haemorrhaging and low (≤1.80*10^− 3^ mm^2^/s for MD, 1.40*10^− 3^ mm^2^/s for *D*_*bi*_ and 1.05*10^− 3^ mm^2^/s for *D*_*tri*_) in tumours with necrosis or sarcomatoid differentiation.

**Conclusion:**

Parameters derived from a two- or three-component fit of the diffusion signal are sensitive to histopathological features of kidney lesions.

**Electronic supplementary material:**

The online version of this article (10.1186/s40644-018-0178-0) contains supplementary material, which is available to authorized users.

## Background

As a result of the increased use of abdominal imaging, more (asymptomatic) small (≤ 4 cm) renal masses are incidentally discovered. In a series of 173 patients only 58% of kidney tumours < 4 cm were malignant, whereas all kidney tumours > 7 cm were [1]. Hence, a substantial amount of incidentally discovered renal masses is not malignant [2–4]. The management of renal lesions includes radical or partial nephrectomy, minimal invasive ablative techniques or active surveillance. Because of concern for chronic kidney disease, nephron sparing surgery is preferred [5, 6] but more importantly unnecessary surgery should be avoided. One way to realize this is by distinguishing between lesion types and reliably diagnosing benign tumour types, such as oncocytoma, prior to treatment [7]. However, with currently available clinical imaging modalities, benign renal masses are indistinguishable from malignant renal masses [4, 8].

Many magnetic resonance imaging (MRI) techniques have been explored as methods to differentiate between benign and malignant renal lesions or between renal cell carcinoma (RCC) subtypes [4, 9–11]. One promising technique is diffusion-weighted imaging (DWI), which allows quantification of water motion in tissues without administration of exogenous contrast materials [12–16]. The apparent diffusion coefficient (ADC), derived from a mono-exponential model, is believed to reflect tissue cellularity as a higher tissue density will amount to more restricted diffusion, hence a lower diffusion value. However, ADC values for different subtypes may overlap, making determination of cut-off values to distinguish between benign and malignant solid renal masses problematic [17].

More complex models of diffusion, such as the diffusion tensor model (DTI) and the intravoxel incoherent motion model (IVIM), allow deriving additional information. DTI-derived parameters fractional anisotropy (FA) and mean diffusivity (MD) have been correlated with histological parameters such as cell density and nuclear grade [18]. The IVIM model is a bi-exponential model that includes molecular diffusion and microcirculation of blood in the capillary network (‘pseudodiffusion’) [19]. A combination of pseudodiffusion fraction *f*_*bi*_ and the perfusion-free diffusion coefficient *D*_*bi*_ from IVIM model is able to differentiate between renal tumour types [20, 21]. Recently, the IVIM model was expanded to a three-component model by adding an additional component that accounts for intermediately fast water motion in the kidney [22, 23]. The aim of this study is to compare parameters obtained from DTI, intravoxel incoherent motion (IVIM), and tri-exponential models of the diffusion signal of kidney lesions, for the characterization of renal lesions. Because tumours are usually not uniform and may consist of several areas with different structural patterns, we compare diffusion parameters with histopathological results.

## Methods

### Subjects

Approval of our institution’s ethical committee was obtained for this prospective study and all subjects provided written informed consent. From March 2016 to May 2017, sixteen patients (11 male, age 65 (range 50–76) years old, 5 female, age 60 (range 48–72), total group: age 64 (range 48–76) years old) who had suspected kidney tumours and were planned to undergo radical or partial nephrectomy based on standard clinical diagnostic criteria were included. After including the first five consecutive patients, patients were also selected on tumour size (≤ 4 cm on radiologic examination) in order to increase chance of including benign solid lesions. After surgical resection of the tumour, kidney tumour type was determined according to the WHO classification of tumours of the urinary system [24] by histopathological examination of 2-μm-thick sections of formalin-fixed and paraffin-embedded tumour tissue blocks using haematoxylin-eosin (HE) staining.

### Scans

A *T*_*2*_ weighted sequence was performed for anatomical reference, followed by a DTI sequence (*b* = 0, 100 and 300 s/mm^2^ in 15 gradient direction) and a DWI sequence including ten *b-*values (*b* = 0, 10, 25, 40, 75, 100, 200, 300, 500 and 700 s/mm^2^ in six gradient directions) on a 3 T MRI clinical scanner (Philips, Ingenia, Philips Healthcare, Best, The Netherlands), see Table 1 for MRI acquisition details.

**Table 1:** MRI acquisition details of the T2-TSE, DTI and IVIM protocols

Sequence	T2-TSE	DTI	IVIM
Respiratory correction	Breath hold	Trigger	Trigger
Scan time per breath hold/ respiration	00:15.6	0:02.8	0:03.0
Acquisition plane	Coronal	Coronal	Coronal
Field of view	450 × 450	336 × 204	336 × 204
TSE factor	20	–	–
TR/TE (ms)	1200/80	2799/47	3007/56
Startup echoes	0	–	–
b-value (s/mm^2^)	–	0, 100, 300	0, 10, 25, 40, 75, 100, 200, 300, 500, 700
Flip angle (deg)	90	–	–
Gradient directions	–	15	6
EPI factor (ETL)	–	61	61
SENSE factor	2	1.5	1.5
Acquisition matrix	412 × 281	112 × 67	112 × 67
Acquisition voxel size (mm^3^)	1.09 × 1.60 × 3.0	3.0 × 3.0 × 3.0	3.0 × 3.0 × 3.0
Half Fourier scan factor	0.599	0.655	0.655
Slice thickness/gap (mm)	3.0 /−	3.0/−	3.0/−
Number of slices	25	30	30
Number of averages	1	1 (b = 0, 6)	1 (b = 0, 4)
Type of fat suppression	No	SPIR	SPIR
Total acquisition time (min, without trigger)	00:33.6	01:51.0	02:57.4

### Data processing

To enable accurate parameter fitting all scans were corrected for (breathing) motion before further processing. Due to differences in motion between the right and left kidneys, they were cropped and processed as separate data sets, as described previously [22]. All pre-processing was performed using diffusion imaging analysis package DTItools [github.com/mfroeling/DTITools] [25] and image registration toolbox Elastix [http://elastix.isi.uu.nl/] [26]. First, *T*_*2*_ scans were processed to correct for slice by slice misalignment due to acquisition in multiple breath-holds using a rigid 2D registration algorithm after being resampled to 2 mm isotropic using a single interpolation method. Finally, all DWI data was corrected for breathing motion, by registering them to the unweighted volume using a rigid 2D b-spline registration algorithm after which the DWI data was registered to the reference *T*_*2*_ scan using a 3D affine registration algorithm [22].

### Parameter maps

From the DTI data the FA and MD were calculated using an iterative weighted linear least squares algorithm with outlier rejection using ExploreDTI [27]. From the IVIM data, bi- and tri-exponential diffusion decay parameters, i.e. the mean diffusion (*D*_*bi*_ for bi-exponential and *D*_*tri*_ for tri-exponential fitting), and the signal fraction attributed to pseudo-diffusion (*f*_*star*_ for bi-exponential and *f*_*fast*_ and *f*_*interm*_ for tri-exponential fitting), were obtained by fitting a two and three-component model to the multiple b-value DWI data, as described previously [22, 23]. To make a comparison between the DTI and IVIM data, the mean diffusion from a mono-exponential fit, D_mono_ was also obtained.

Regions of interest (ROIs) to segment the tumour volumes were manually defined on the combined *T*_*2*_ and DWI data by the principal researcher (S.v.B., 4 years of experience) in agreement with an experienced radiologists (C.K., 12 years of experience) using image segmentation toolbox ITK snap [28]. ROIs were placed inside the tumour, rather than following the contour, to limit the contribution of the signal from other tissue types due to partial volume effect or imprecise image registration. For comparison of tumour tissue with healthy kidney parenchyma, the cortex and medulla in the healthy contralateral kidneys were segmented using an automated algorithm as in [22]. The mean and standard deviation of the diffusion parameters were obtained for healthy cortex and medulla and lesion ROIs. Parameter maps MD, *D*_*bi*_, *D*_*tri*_, *f*_*star*_, *f*_*fast*_ and *f*_*interm*_ were obtained for visual comparison with *T*_*2*_ and (if available) photographs of the gross appearance of the resected kidney tumours before histological examination.

### Statistical analysis

All statistical tests were performed using SPSS (version 23.0. Armonk, NY: IBM Corp.). Healthy cortex and medulla were compared with all solid lesions using a Wilcoxon Signed Ranks test. The means of parameters MD, FA, *D*_*bi*_, *D*_*tri*_, *f*_*star*_, *f*_*fast*_ and *f*_*interm*_ in healthy cortex and medulla, different types of RCCs, cysts and benign solid lesions were compared using multivariate analysis of variance (MANOVA). Bonferroni correction was applied, and a *p*-value < 0.05 was considered significant for all statistical tests.

## Results

### Subjects and scans

**Fig. 1:**
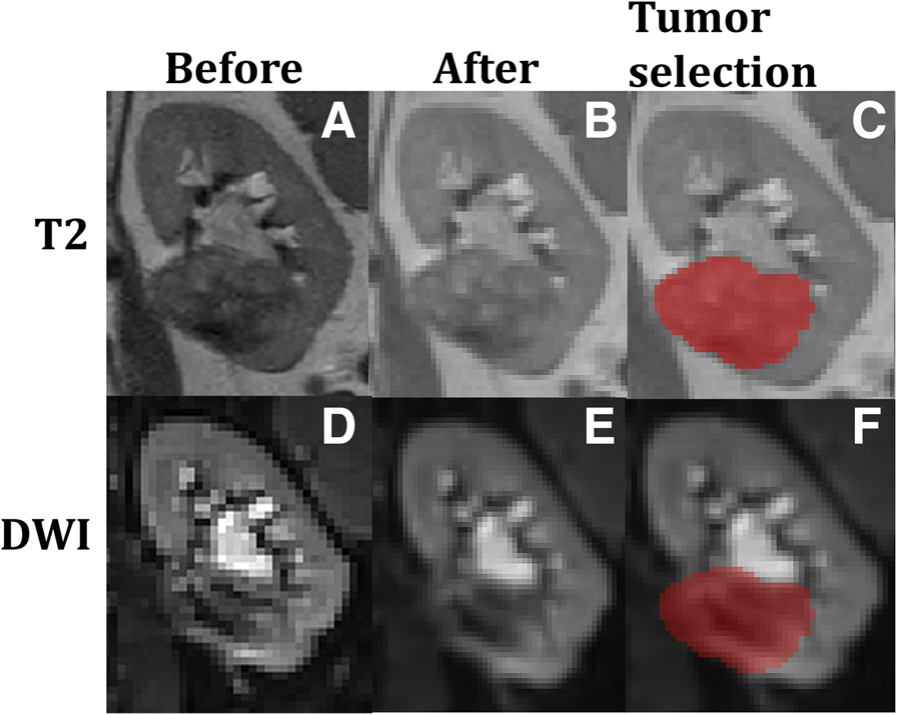
Anatomical reference *T*_*2*_ image before (**a**) and after (**b**) motion correction and image registration diffusion weighted images before (**d**) and after (**e**) processing, ROI selecting tumor tissue in *T*_*2*_ (**c**) and same ROI projected on DWI (**f**)

All patients were successfully scanned. One scan was removed before processing due to artefacts resulting from a lower-back implantation. After visual inspection following processing two other scans were removed, due to poor motion correction results and an error in the data. In the remaining thirteen scans, thirteen solid lesions (average size of maximum diameter, determined by histopathological examination 3.85 cm, range 0.8–7.5 cm) and five fluid-filled cysts were found. Examples of the raw acquired data and the data after motion correction and image registration together with the ROI placement in one lesion are shown in Fig. 1.

### Histological examination

**Fig. 2:**
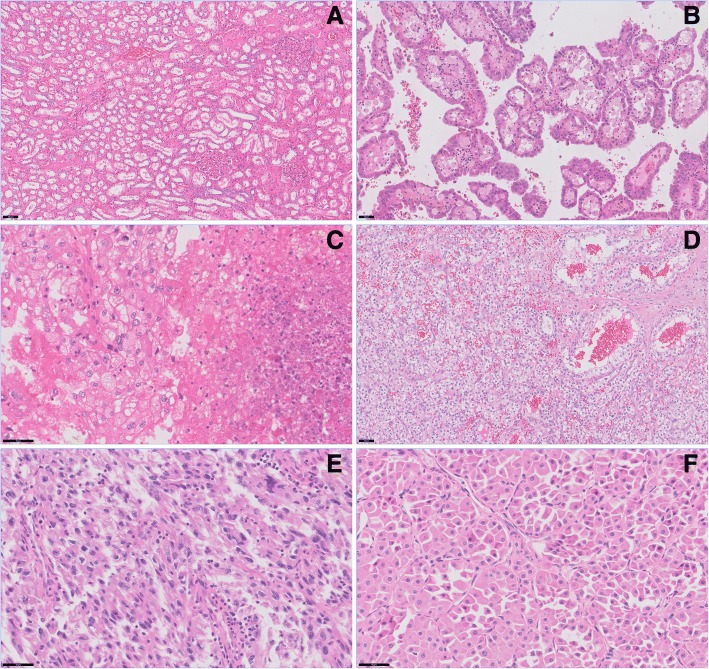
Histopathological features of kidney (tumor) tissue types, sections are stained with haematoxylin-eosin. **a** Normal kidney tissue, with kidney cortex including glomeruli on the right and medulla including tubular structures to the left. Magnification: 100x, scale bar represents 100 μm, (**b**) Papillary RCC which presents with papillae and the presence of macrophages in nuclei. Magnification: 200x, (**c**) Clear cell RCC with to the right a necrotic area. Magnification: 400x, (**d**) Clear cell RCC with to the right micro cystic structures. Magnification: 200x,, (**e**) Sarcomatoid differentiation in RCC, displaying elongated, spindle-shaped cells, high cellularity and cellular atypia. Magnification: 400x, (**f**) Oncocytoma, displaying granular eosinophilic cytoplasm. Magnification: 400x scale bar represents 50 μm in (**b**-**f**)

Of the solid lesions, eleven were considered to be malignant (nine clear cell RCCs (cc-RCCs), two papillary cell RCCs (p-RCCs) and two were considered to be benign (one haemangioma of the kidney capsule and one oncocytoma). Of the nine cc-RCCs, only one had a homogeneous microstructural pattern consisting mainly of clear cells. Others had considerable amounts of necrosis, sarcomatoid differentiation, haemorrhaging or deviating growth patterns such as papillary, tubular or cystic growth. In Fig. 2, histopathological features of several kidney tumour tissues are displayed.

### Parameter maps

**Fig. 3:**
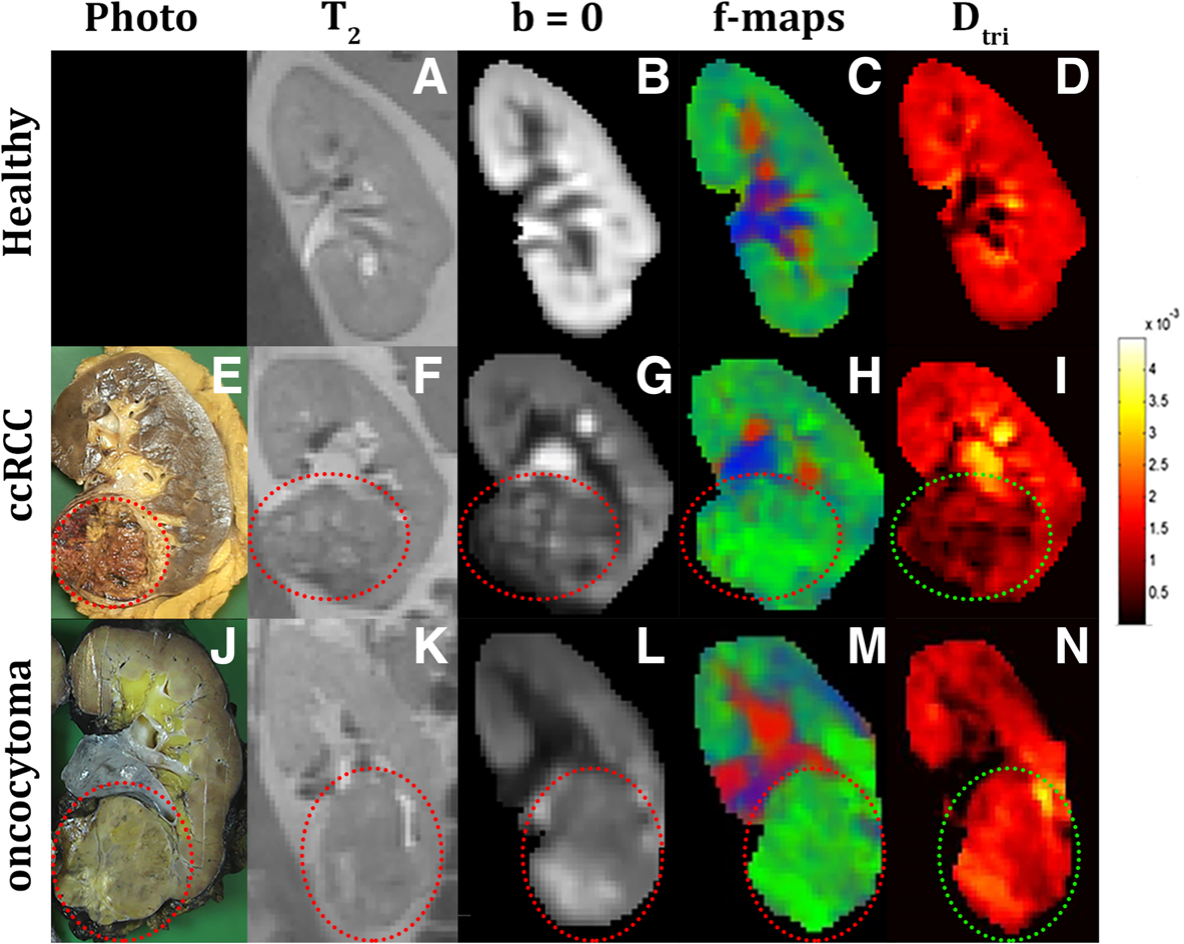
Diffusion-derived parameter maps of an unaffected kidney (**a**-**d**), a kidney with renal cell carcinoma (**e**-**h**), and a kidney with oncocytoma (**i**-**l**). First row (**e**, **j**): gross appearance of the whole kidney or tumour after nephrectomy, (**a**, **f**, **k**): anatomical reference (after processing) (T_2_-TSE), second row (**b**, **g**, **l**): the unweighted image of the diffusion scan after motion correction and image registration (DWI-b0), third row (**c**, **h**, **m**): a merge of the fraction maps from the tri-exponential fit red = *f*_*fast,*_ blue = *f*_*interm*,_ green = *f*_*slow*_ (1- *f*_*interm*_ - *f*_*fast*_), fourth row (**d**, **i**, **n**): diffusion coefficient from the tri-exponential fit, (*D*_*tri*_)

Figure 3 shows MRI data *(T*_*2*_ and DWI b = 0) parameters maps and (where available) photographs of the gross appearance of the resected kidney tumours for: a contralateral unaffected kidney (A-D), a case of cc-RCC (E-H) and the oncocytoma (I-L). In the Additional file 1: Figure S1 the gross appearance, MRI data and parameter maps of all tumour types are shown. The fractions of the diffusion components are shown as merged *f*-maps where *f*_*fast*_, *f*_*interm*_ and *f*_*slow*_ are colour coded red, blue and green, respectively. For the unaffected kidney, *f*_*fast*_ (red) was high in those areas with a high blood flow (e.g. large blood vessels) whereas *f*_*interm*_ (blue) was high in areas with free water (e.g. the pyelum). The diffusion coefficient (*D*_*tri*_) was homogeneous throughout healthy kidney parenchyma.

Upon visual examination, in the maps obtained in a cc-RCC, *D*_*tri*_ was lower throughout the tumour, and *f*_*slow*_ had a high contribution. For the oncocytoma, the lesion did not seem much different from normal kidney parenchyma, although *f*_*slow*_ and *D*_*tri*_ appeared higher.

In the p-RCC, the merged *f*-maps showed a small contribution from *f*_*fast*_ and a larger contribution from *f*_*slow*_. The *D*_*tri*_ map indicated a low diffusion coefficient. In the cc-RCC with sarcomatoid differentiation and the cc-RCC with papillary growth the photographs and the diffusion parameter maps showed a more heterogeneous make-up, indicating a more complex tumour. In the cysts, *f*_*slow*_ had a high contribution and *D*_*tri*_ was high. For the haemangioma, *f*_*slow*_ and *D*_*tri*_ appeared higher.

### Parameter analysis

The mean and standard deviations of parameter values of grouped lesions and measurements of healthy cortex and medulla are given in Table 2. In Additional file 2: Figure S2 *D*_*mono*_ is plotted against *MD* for each lesion. In Additional file 3: Table S1, the values for *D*_*mono*_ are displayed together with the other diffusion coefficients *MD*, *D*_*bi*_*,* and *D*_*tri*_. *MD* and *D*_*mono*_ have a similar order of magnitude for each (group of) lesion, whereas *D*_*bi*_ and *D*_*tri*_ are structurally lower.

**Table 2:** Mean (standard differentiation) of the diffusion parameters obtained from DTI, and the bi- and tri-exponential models for each of the segmented tissue types

	DTI		Bi-exponential model		Tri-exponential model		
	MD [10^− 3^ mm^2^/s]	FA	f_star_ [%]	D_bi_ [10^− 3^ mm^2^/s]	f_fast_ [%]	f_interm_ [%]	D_tri_ [10^− 3^ mm^2^/s]
Healthy Cortex (*n* = 13)	2.16 (0.12)	0.38 (0.09)	10.1 (2.58)	1.93 (0.10)	4.14 (1.92)	28.8 (5.09)	1.41 (0.09)
Healthy Medulla (*n* = 13)	2.21 (0.14)	0.39 (0.08)	9.69 (2.90)	2.02 (0.11)	4.57 (1.74)	26.4 (6.65)	1.55 (0.12)
All solid lesions (*n* = 13)	1.94 (0.32)	0.47 (0.11)	11.6 (3.88)	1.71 (0.43)	7.30(3.29)	18.7 (5.02)	1.39 (0.35)
RCC (*n* = 11)	1.90 (0.32)	0.46 (0.10)	11.6 (3.45)	1.65 (0.40)	7.21 (2.88)	18.3 (5.35)	1.34 (0.33)
Cyst (*n* = 5)	3.04 (0.17)	0.37 (0.11)	1.88 (1.60)	2.90 (0.11)	1.18 (1.70)	8.31 (2.02)	2.74 (0.08)
Benign (*n* = 2)	2.18 (0.28)	0.48 (0.19)	11.6 (7.83)	2.04 (0.58)	7.76 (6.75)	20.9 (2.43)	1.68 (0.45)
Significance^a^	a, b, d, e, g, i x, y, z		b, e, g, i	b, d, e, g, i y, z	a, g, i x, y	a, d, e, g, x, y	b, e, g, i, z

a *p*-value smaller than 0.05 is considered significant

*RCC* renal cell carcinoma, *MD* mean diffusivity, *FA* fractional anisotropy

^a^a = healthy cortex vs. RCC, b = healthy cortex vs. cyst, c = healthy cortex vs. benign

d = healthy medulla vs. RCC, e = healthy medulla vs. cyst, f = healthy medulla vs. benign

g = RCC vs. cyst, h = RCC vs. benign, i = benign vs. cyst

j = healthy cortex vs. healthy medulla

x = healthy cortex vs. all lesions (Wilcoxon Signed Ranks Test), y = healthy medulla vs. all lesions (Wilcoxon Signed Ranks Test)

z = healthy cortex vs. healthy medulla (Wilcoxon Signed Ranks Test)

Solid lesions had a significantly lower MD (1.94 ± 0.32 10^− 3^ mm^2^/s) than healthy tissue (2.16 ± 0.12 10^− 3^ mm^2^/s for cortex, *p* = 0.019 and 2.21 ± 0.14 10^− 3^ mm^2^/s for medulla, *p* = 0.009) and a significantly lower (*p* = 0.023) *D*_*bi*_ (2.02 ± 0.11 10^− 3^ mm^2^/s) than healthy medulla (1.71 ± 0.43 10^− 3^ mm^2^/s), but no significant difference in *D*_*tri*_. MD, *D*_*bi*_ and *D*_*tri*_ were all significantly higher (*p* < 0.002) in cysts than healthy tissue and other lesions (see Table 2).

Compared to healthy tissue (0.38 ± 0.09 for cortex and 0.39 ± 0.08 for medulla), FA was higher in all solid lesions (0.47 ± 0.11), RCCs (0.46 ± 0.10) and benign solid lesions (0.48 ± 0.19) but similar in cysts (0.37 ± 0.11), however these differences were not significant.

*f*_*star*_ did not show significant differences between healthy tissue (10.1 ± 2.58% for cortex, 9.69 ± 2.90% % for medulla), and solid lesions (11.6 ± 3.88%). *f*_*interm*_ was significantly higher in healthy tissue (28.8 ± 5.09% for cortex and 26.4 ± 6.65% for medulla) than solid lesions (18.7 ± 5.02%, *p* = 0.003 for cortex, *p* = 0.011 for medulla) and RCCs (18.3 ± 5.35%, *p* = 0.000 for cortex, *p* = 0.009 for medulla). *f*_*fast*_ was a significantly lower for healthy tissue (4.14 ± 1.92% for cortex, *p* = 0.009, and 4.57 ± 1.74% for medulla, *p* = 0.033) than all solid lesions (7.30 ± 3.29%), and healthy cortex had a significantly lower *f*_*fast*_ than RCCs (7.21 ± 2.88%, *p* = 0.034). Cysts had a significantly lower *f*_*star*_ (1.88 ± 1.60%) than healthy tissue (*p* = 0.000 for cortex, 0.001 for medulla), RCCs (11.6 ± 3.45%, *p* = 0.000) and benign lesions (11.6 ± .83%, *p* = 0.009), a significantly lower *f*_*interm*_ (8.31 ± 2.02%, *p* = 0.034) than RCCs and a significantly lower *f*_*fast*_ (1.18 ± 1.70%) than RCCs (*p* = 0.001) and benign solid lesions (7.76 ± 6.75%, *p* = 0.030).

Parameter values separated according to tumour type are given in Table 3, together with values for individual heterogeneous RCCs. There was a large variation in all parameters among the cc-RCCs. Diffusion coefficients were high (≥ 2.0*10^− 3^ mm^2^/s for MD, 1.90*10^− 3^ mm^2^/s for *D*_*bi*_ and 1.6*10^− 3^ mm^2^/s for *D*_*tri*_) in cc-RCCs with cystic structures (see Fig. 2d for microscopic photograph) and/or haemorrhaging and low (≤ 1.80*10^− 3^ mm^2^/s for MD, 1.40*10^− 3^ mm^2^/s for *D*_*bi*_ and 1.05*10^− 3^ mm^2^/s for *D*_*tri*_) in tumours with necrosis (Fig. 2c) or sarcomatoid differentiation (Fig. 2e). *f*_star_ and *f*_*fast*_ is high (16.62 and 12.43% respectively) in a tumour with extensive haemorrhaging, and low (≤ 11% for *f*_star_ and ≤ 6.5% for *f*_*fast*_) in tumours with cystic structures and/or necrosis. *f*_*interm*_ is particularly high in a cc-RCC with micro-cystic structures and haemorrhaging.

**Table 3:** Mean (standard differentiation) of the diffusion parameters obtained from DTI, and the bi- and tri-exponential models for each type of solid lesion

	DTI		Bi-exponential model		Tri-exponential model		
	MD [10^−3^ mm^2^/s]	FA	f_star_ [%]	D_bi_ [10^−3^ mm^2^/s]	f_fast_ [%]	f_interm_ [%]	D_tri_ [10^−3^ mm^2^/s]
cc-RCC (*n* = 9)	1.94 (0.33)	0.46 (0.12	11.6 (3.62)	1.71 (0.42)	7.36 (3.14)	18.8 (5.78)	1.38 (0.34)
cc-RCC with sarcoid differentiation, extensive necrosis and hemorrhaging (*n* = 1)	1.77	0.58	12.5	1.38	7.83	20.1	1.04
cc-RCC with extensive necrosis (*n* = 1)	1.19	0.64	10.78	0.92	6.13	15.11	0.71
cc-RCC with papillary growth (*n* = 1)	1.82	0.28	16.5	1.49	11.2	20.6	1.19
cc-RCC with cells situated in nests and extravasation of erythrocytes (*n* = 1)	1.94	0.40	14.29	1.71	9.11	22.25	1.71
cc-RCC with areas of low cell density, hemorrhaging and cystic structures (*n* = 1)	2.24	0.57	10.35	1.90	6.22	12.46	1.68
cc-RCC with extensive hemorrhage (*n* = 1)	2.01	0.46	16.62	1.71	12.43	16.09	1.49
cc-RCC with cystic and solid areas (*n* = 1)	2.20	0.45	8.69	1.97	6.25	15.31	1.69
cc-RCC with micro- cystic structures and hemorrhaging (*n* = 1)	2.17	0.36	7.95	2.39	2.83	31.72	1.68
p-RCC (*n* = 2)	1.68 (0.20)	0.50 (0.01)	11.3 (3.8)	1.36 (0.06)	6.56 (1.80)	16.3 (2.88)	1.14 (0.06)
Hemangioma (*n* = 1)	2.38	0.61	6.06	2.45	2.99	22.6	1.99
Oncocytoma (*n* = 1)	1.98	0.34	17.1	1.63	12.5	19.2	1.36

*cc-RCC* clear cell renal cell carcinoma, *p-RCC* papillary renal cell carcinoma, *MD* mean diffusivity, *FA* fractional anisotropy

The haemangioma had the highest diffusivity in all models (2.38*10^− 3^ mm^2^/s for MD, 2.45*10^− 3^ mm^2^/s for *D*_*bi*_ and 1.99*10^− 3^ mm^2^/s for *D*_*tri*_). Additionally, the haemangioma had the highest FA (0.61). The oncocytoma (Fig. 2f) had the highest *f*_star_ (17.1%) and *f*_*fast*_ (12.5%), although the cc-RCC with papillary growth had a comparable *f*_star_ (16.5%). The cysts and haemangioma had a low *f*_star_ (7.95 and 6.06%, respectively) and *f*_*fast*_ (2.83 and 2.99% respectively). *f*_*interm*_ was in the range of 19–23% except for p-RCC (16%) and cysts (32%).

**Fig. 4:**
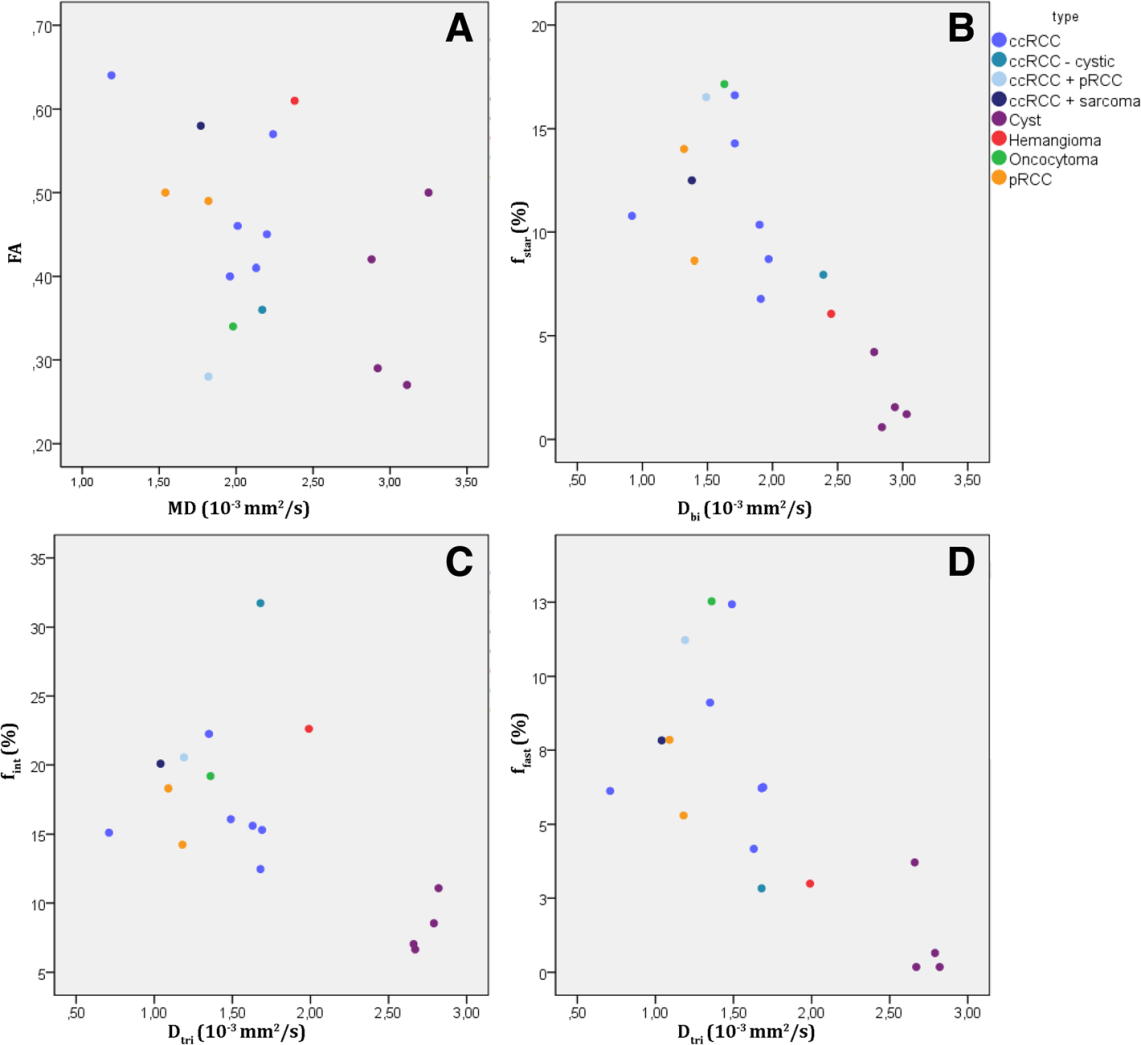
scatter plots, each point represents a single lesion. **a**: FA vs. MD (DTI fit), **b**: *D*_*bi*_ vs. *f*_*star*_ (bi-exponential fit). **c**: *D*_*tri*_ vs. *f*_interm_ (tri-exponential fit). **d**: *D*_*tri*_ vs. *f*_*fast*_ (tri-exponential fit). RCC = renal cell carcinoma, cc-RCC = clear cell renal cell carcinoma, p-RCC = papillary renal cell carcinoma

In Fig. 4, each lesion is represented by a dot in the scatter plot, plotting DTI parameters MD versus FA (Fig. 3a), IVIM parameters *D*_*bi*_ versus *f*_*star*_ (Fig. 3b), three-component parameters *D*_*tri*_ versus *f*_*interm*_ (Fig. 3c) and *D*_*tri*_ versus *f*_*fast*_ (Fig. 3d). Cysts were recognizable by a high MD, *D*_*bi*_ and *D*_*tri*,_ but they were more grouped in *D*_*tri*_ versus *f*_*interm*_/*f*_*fast*_ due to a consistently low *f*_*interm*_ and *f*_*fast.*_ In *D*_*bi*_ versus *f*_*star*_ and *D*_*tri*_ versus *f*_*fast*_ several general groupings were identifiable; the cysts are located in the lower right corner, the cc-RCCs in the middle and p-RCC (Fig. 2b for microscopic photograph) in the middle to the left. The oncocytoma is located above the cc-RCCs and the haemangioma between cc-RCCs and the cysts. However, one of the cc-RCCs is closely located to the oncocytoma due to a high value of *f*_*star*_ and *f*_*fast*_. The p-RCCs and cc-RCC with sarcomatoid differentiation seemed to have a slightly lower *D*_*bi*_ and *D*_*tri*_ than most cc-RCCs, but one cc-RCC has a much lower diffusivity than all other lesions.

**Fig. 5:**
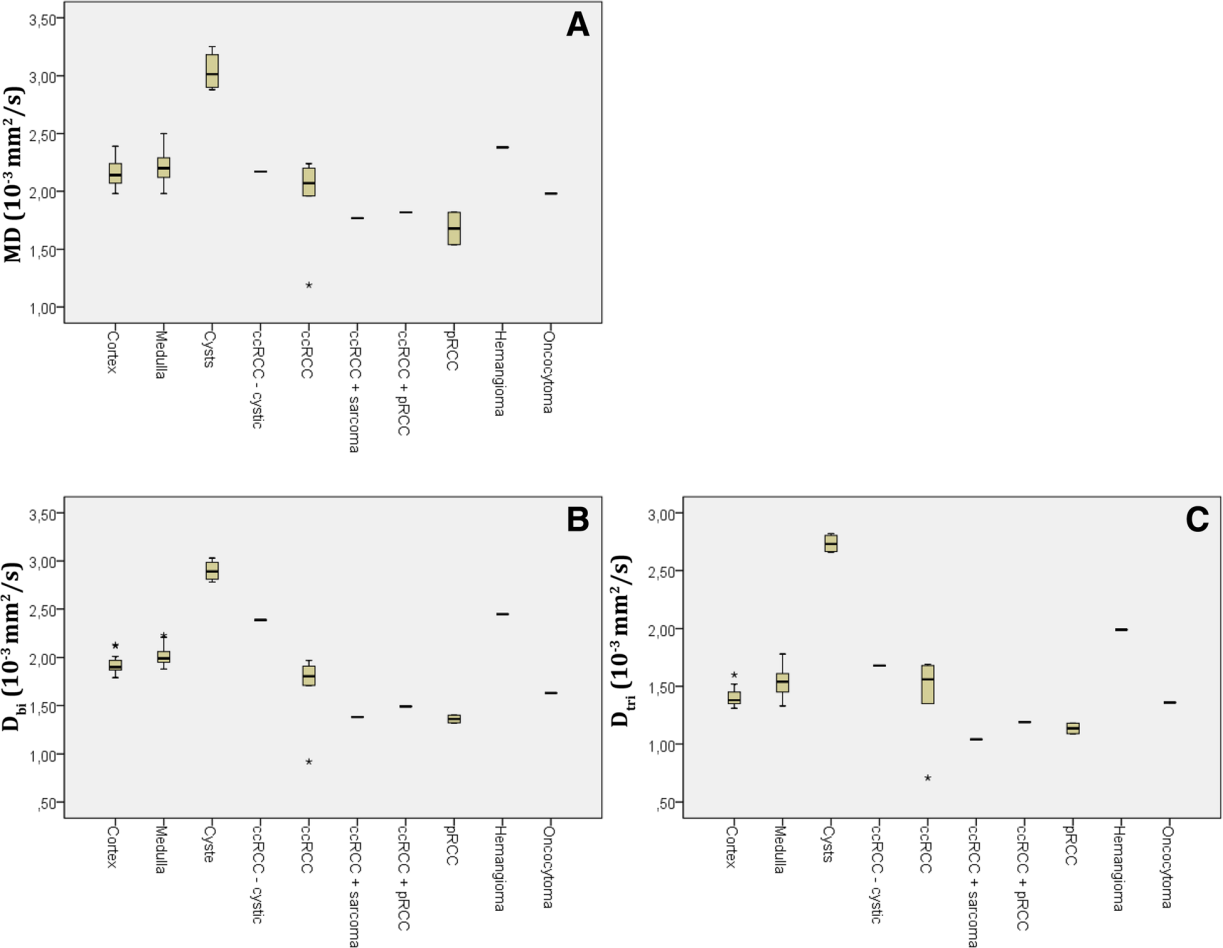
Boxplots for diffusion coefficients, (**a**) MD, (**b**) *D*_*bi*_, and (**c**) *D*_*tri*_, for individual lesions, cysts, RCCs and healthy cortex and medulla. Points represent individual lesions, error bars groups of lesions. RCC = renal cell carcinoma, cc-RCC = clear cell renal cell carcinoma, p-RCC = papillary renal cell carcinoma

**Fig. 6:**
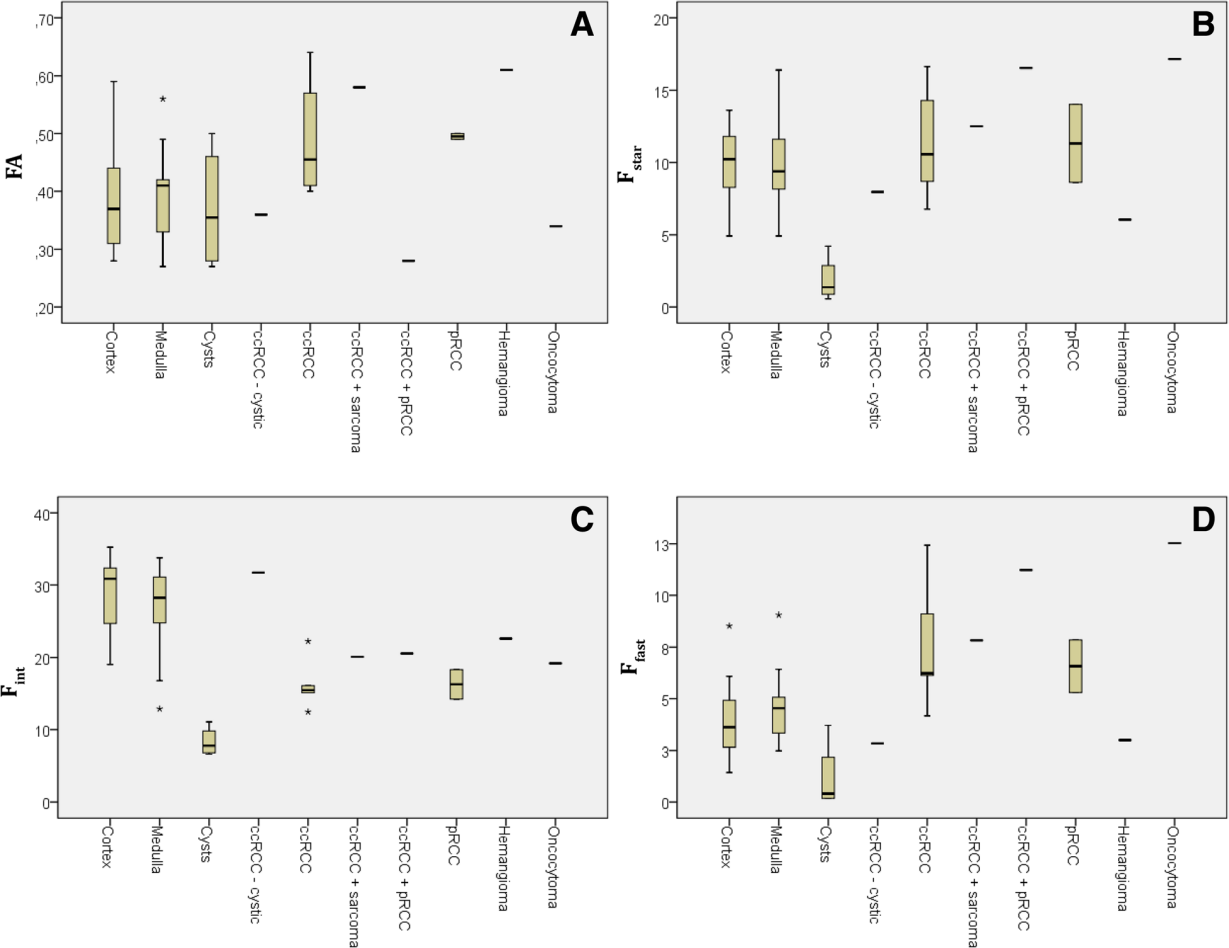
Boxplots for (**a**) FA, (**b**) *f*_*star,*_ (**c**) *f*_*fast*_*,* and (**d**) and *f*_*interm*_ or individual lesions, cysts, RCCs and healthy cortex and medulla. Points represent individual lesions, error bars groups of lesions. RCC = renal cell carcinoma, cc-RCC = clear cell renal cell carcinoma, p-RCC = papillary renal cell carcinoma

Figures 5 and 6 show box plots for MD (Fig. 5a), *D*_*bi*_ (Fig. 5b), *D*_*tri*_ (Fig. 5c), FA (Fig. 6a), *f*_*star,*_ (Fig. 6b), *f*_*fast*_ (Fig. 6c) and *f*_*interm*_ (Fig. 6d). The *f*_*interm*_ and *f*_*fast*_ show more pronounced differences and less overlap between individual lesions than *f*_*star.*_ and compared to MD, both *D*_*bi*_ and *D*_*tri*_ gave more pronounced differences and less overlap between tissue types.

## Discussion

In this study, we have compared the DTI, IVIM and three-compartment models of the diffusion signal for the characterization of renal lesions. In the study population of sixteen patients who received radical or partial nephrectomy, two lesions were found benign upon histological examination. This indicates that resection was unnecessary and could have been prevented if these lesions were identified as non-malignant prior to surgery. Based on our results the haemangioma could potentially have been identified using diffusion-derived parameters, notably, a high *D*_*bi*_ and *D*_*tri*_ and a low *f*_*star*_ and *f*_*fast*_.

Previous studies applying DWI to evaluate kidney tumour type concluded that kidney lesions generally have a lower ADC than normal kidney tissue [13, 17, 29] and some studies also found a higher mean ADC value in benign lesions than in malignant lesions [15, 30, 31]. Studies typically find a higher ADC in oncocytomas than in RCCs [12–15, 17, 30–32] and therefore, ADC is likely to be a valuable parameter in evaluating tumour type. However, in itself it is not sufficient as a clinical index due to overlap in values between tumour tissue types [16, 17, 33].

In line with previous studies MD in this study was significantly lower in solid lesions than in healthy tissue, and RCCs had a lower MD than benign lesions and cysts. Differences in diffusivity between tissue types are usually attributed to differences in tissue cellularity, a higher cellularity will result in more restricted diffusion and therefore, more aggressive lesions are expected to present with a lower diffusivity [15, 21]. Our study also showed similar results: cysts had the highest diffusion coefficients whereas lesions with a high degree of necrosis had low diffusivity, which we have assigned to an increase in with a diffusion-restricting elements, such as macromolecules, and disorganisation.

In the two-component IVIM model, a fast moving component is separated from the diffusion signal, resulting in a lower diffusion coefficient *D*_*bi*_. The fraction *f*_*star*_ of the diffusion signal that is attributed to fast moving water has been correlated to renal tumour vascularity [21]. As in this study, a lower MD and *D*_*bi*_ for p-RCC than cc-RCC and similar values for *f*_*star*_ in p-RCCs were found previously [21]. A tri-exponential fit of the diffusion signal in the kidney was previously shown to provide additional information on structures associated with pseudodiffusion by separating the fast from intermediate pseudodiffusion resulting in signal fractions *f*_*interm*_ associated with a diffusion rate in the order of magnitude of free water and *f*_*fast*_ associated with perfusion [22, 23], and comparable values to this study for parameters derived from DTI, two- and three-compartment fitting for healthy cortex and medulla were reported [22].

MD was the only diffusion coefficient to be significantly different in healthy tissue from RCCs but within the tumour type groups the MD range was wider whereas the differences between tumour types were more pronounced in *D*_*bi*_ and *D*_*tri,*_. Because diffusion coefficients *D*_*bi*_ and *D*_*tri*_ exclude the fraction of the diffusion signal that is attributed to fast water movement, they were lower but more precise than MD. Therefore, *D*_*bi*_ and *D*_*tri*_ better reflect tissue diffusion, making both these parameters more specific for tissue cellularity. However, none of the diffusion coefficients could be used to reliably distinguish between lesion types, as diffusion coefficients overlap. For a better contrast between lesion types, tissue cellularity (MD, *D*_*bi*_ or *D*_*tri*_) can be combined with a measure for vascularisation (*f*_*star*_ or *f*_*fast*_). For example, a combination of *D*_*bi*_ and *f*_*star*_ was previously shown to discriminate between renal tumour subtypes [21].

There was a large variation in diffusion parameter values between individual cc-RCC tumours due to tumour heterogeneity and differences in microstructural make-up. In addition, drawing statistical inferences from this study is limited due to the small number of cases and the sensitivity of the analysis to outliers. Therefore we have also analysed the results from individual lesions, associating histopathological characteristics with the interpretations of diffusion parameters outlined above. In this analysis, low diffusion coefficients were found in cc-RCCs with a high tissue density (due to extensive necrosis or sarcomatoid differentiation) whereas high diffusion coefficients were found in cc-RCCs with cystic structures. Additionally, tumours with a high perfusion rate are characterized by a high value of *f*_*star*_ or *f*_*fast*_ whereas tumours with a low perfusion rate, such as the haemangioma, cysts and the cc-RCCs with cystic structures have lower *f*_*star*_ or *f*_*fast*_*.* Hence, these diffusion parameters seem to be indicative of histopathological features of kidney tumours.

Although both *f*_*star*_ and *f*_*fast*_ seem to correlate to vascularisation, only the three-component parameters *f*_*fast*_ and *f*_*interm*_ show significant differences between different tissues whereas two-component parameter *f*_*star*_ does not, showing that the tri-exponential model provides additional information over the bi-exponential model.

Because of the limited amount of cases it is impossible to formulate conclusions regarding the characterisation of kidney tumour type. Therefore, we have also analysed individual lesions relating our findings to histopathological details. The initial results from this analysis indicate that diffusion parameters are sensitive to histopathological features of kidney lesions, which is a first step towards non-invasive characterisation of these lesions prior to treatment. An improvement to this study would be to spatially correlate histopathology to parameter measurements and maps [34]. This would result in more specific validation of diffusion parameters, confirming the correlation between diffusion parameters to histopathological features of tissues, such as cellularity, perfusion and cystic structures. In addition, to establish what parameter values should be used to confidently distinguish between benign and malignant lesions and draw statistically significant conclusions, a larger study population should be included. However, since kidney tumour type is unknown before histopathological evaluation, researchers have no control over which tumour types are included. To increase the amount of benign kidney tumours, only small (≤ 4 cm) renal masses (about 40% benign [1]) can be included. Additionally, this study shows that parameters derived from the DTI sequence (MD and FA) do not provide additional information over parameters derived from a multiple *b-*value sequence (from two- and three- component fits). Hence, the DTI sequence can be omitted, decreasing total scanner time with one third, to about 30 min.

## Conclusion

In conclusion, parameters derived from a two- or three-component fit of the diffusion signal are sensitive to histopathological features of kidney lesions.

## Additional files

Additional file 1: **Figure S1.** Diffusion-derived parameter maps of each tumor type: an unaffected kidney (A-D), RCC (E-I), clear cell renal cell carcinoma (cc-RCC) with sarcomatoid differentiation (J-N), papillary cell clear cell carcinoma (O-S), cc-RCC with papillary growth (T-X), hemangioma (Y-Ba), simple cyst (Ca-Fa), RCC with micro cysts (Ga-Ka), oncocytoma (La-Pa). First row: gross appearance of the whole kidney or tumor after nephrectomy, second row: anatomical reference (after processing) which is used to manually draw a mask of the whole kidney and tumor (T_2_-TSE), third row: the unweighted image of the diffusion scan after processing and masking (DWI-b0), fourth row: a merge of the fraction maps from the tri-exponential fit, red = *f*_*fast,*_ blue = *f*_*interm*,_ green = *f*_*slow*_ (1- *f*_*interm*_ - *f*_*fast*_), fifth row: diffusion coefficient from the tri-exponential fit (*D*_*tri*_). (TIF 36317 kb)

Additional file 2: **Figure S2.** *D*_*mono*_ plotted against other diffusion coefficients *MD* (A), *D*_*bi*_ (B) and *D*_*mono*_ (C) for each lesions. *MD* versus *D*_*mono*_ displays good correlation, whereas *D*_*bi*_ and *D*_*tri*_ are structurally lower. Cor-heal = healthy cortex, med-heal = healthy medulla, ccRCC = clear cell renal cell carcinoma, pRCC = papillary cell Rhema = haemangioma, onco = oncocytoma, RCC, ccRCC-pRCC = ccRCC with papillary growth, ccRCC-sarc = ccRCC with sarcomatoid differentiation, ccRCC-cyst is ccRCC with micro-cystic structures. (TIF 1288 kb)

Additional file 3: **Table S1.** Comparison of diffusion coefficients MD, D_mono_, D_bi,_ D_tri_. (PDF 682 kb)

## Abbreviations

ADC: Apparent diffusion coefficient
cc-RCC: Clear cell renal cell carcinoma
DTI: Diffusion tensor imaging
DWI: Diffusion weighted imaging
FA: Fractional anisotropy
HE: Haematoxylin-eosin
IVIM: Intravoxel incoherent motion
MD: Mean diffusivity
p-RCC: Papillary cell renal cell carcinoma
RCC: Renal cell carcinoma
ROI: Region of interest
WHO: World Health Organisation
