# Supplementary figures and images for: Mono, bi- and tri-exponential diffusion MRI modelling for renal solid masses and comparison with histopathological findings
Source: Cancer Imaging. 2018 Nov 26;18:44. doi: 10.1186/s40644-018-0178-0 (PMC6260899; doi:10.1186/s40644-018-0178-0)

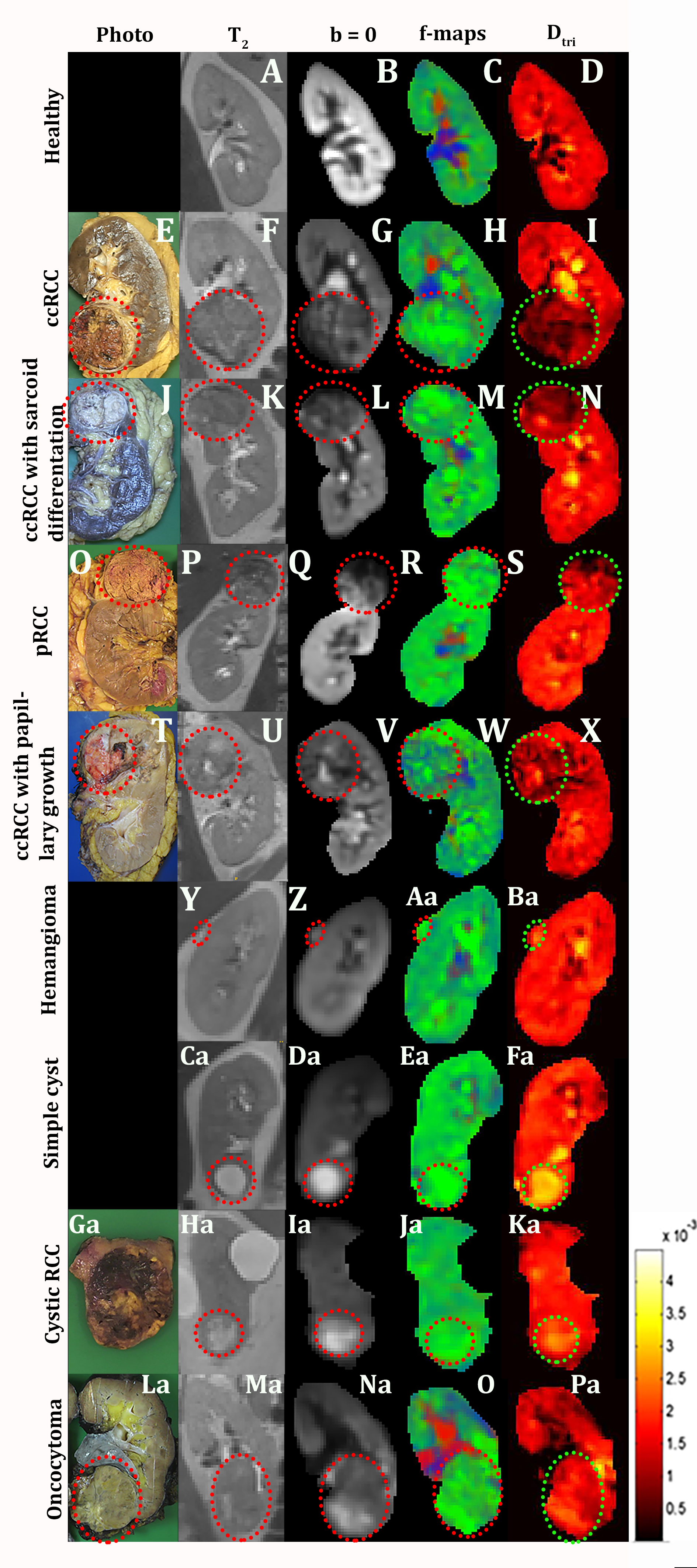

Supplement: Supplementary file 1 — Figure S1. Diffusion-derived parameter maps of each tumor type: an unaffected kidney (A-D), RCC (E-I), clear cell renal cell carcinoma (cc-RCC) with sarcomatoid differentiation (J-N), papillary cell clear cell carcinoma (O-S), cc-RCC with papillary growth (T-X), hemangioma (Y-Ba), simple cyst (Ca-Fa), RCC with micro cysts (Ga-Ka), oncocytoma (La-Pa). First row: gross appearance of the whole kidney or tumor after nephrectomy, second row: anatomical reference (after processing) which is used to manually draw a mask of the whole kidney and tumor (T2-TSE), third row: the unweighted image of the diffusion scan after processing and masking (DWI-b0), fourth row: a merge of the fraction maps from the tri-exponential fit, red = ffast, blue = finterm, green = fslow (1- finterm - ffast), fifth row: diffusion coefficient from the tri-exponential fit (Dtri). (TIF 36317 kb) [file 40644_2018_178_MOESM1_ESM.tif]

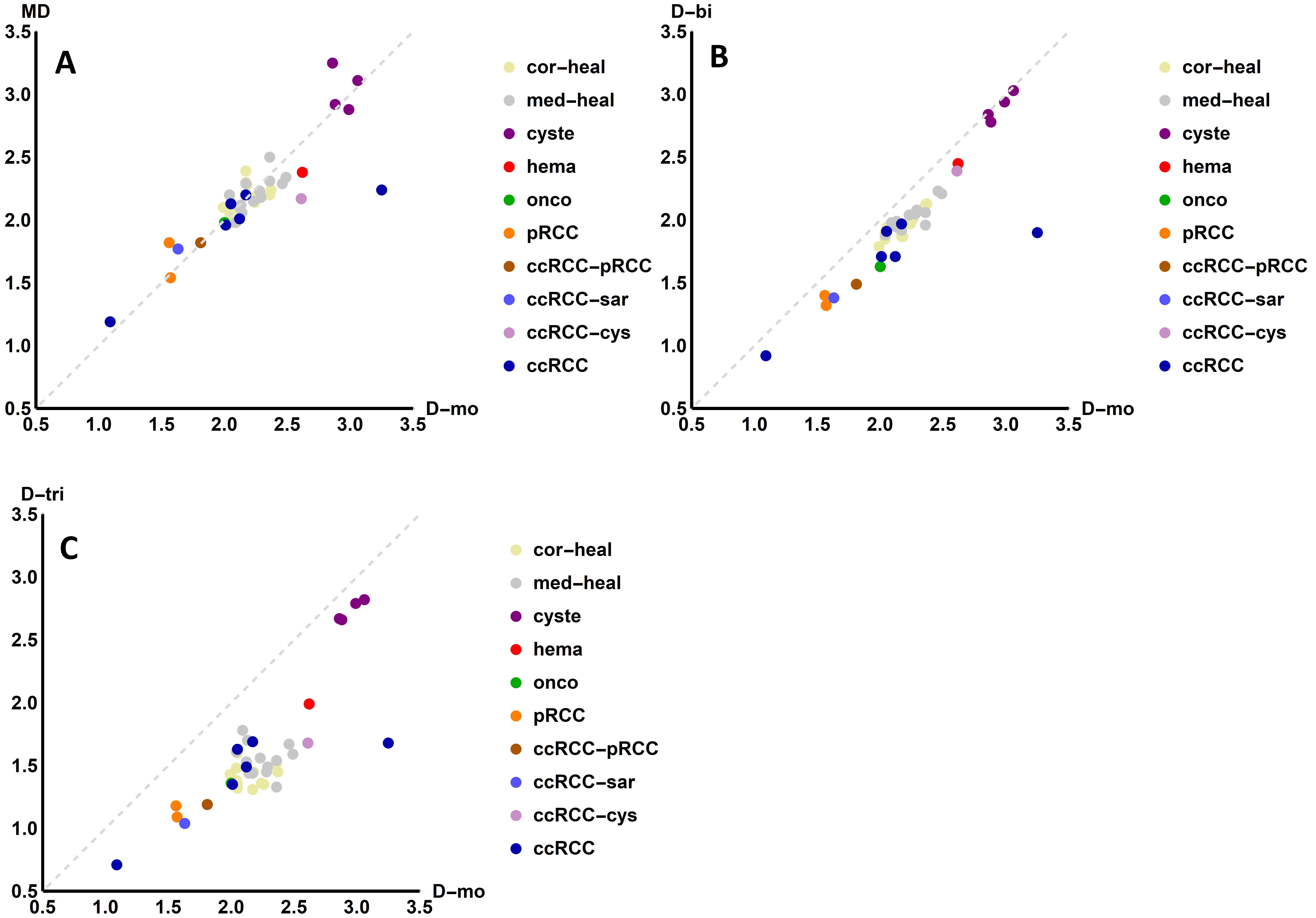

Supplement: Supplementary file 2 — Figure S2. Dmono plotted against other diffusion coefficients MD (A), Dbi (B) and Dmono (C) for each lesions. MD versus Dmono displays good correlation, whereas Dbi and Dtri are structurally lower. Cor-heal = healthy cortex, med-heal = healthy medulla, ccRCC = clear cell renal cell carcinoma, pRCC = papillary cell Rhema = haemangioma, onco = oncocytoma, RCC, ccRCC-pRCC = ccRCC with papillary growth, ccRCC-sarc = ccRCC with sarcomatoid differentiation, ccRCC-cyst is ccRCC with micro-cystic structures. (TIF 1288 kb) [file 40644_2018_178_MOESM2_ESM.tif]
