# Supplementary material for: Mono, bi- and tri-exponential diffusion MRI modelling for renal solid masses and comparison with histopathological findings
Source: Cancer Imaging. 2018 Nov 26;18:44. doi: 10.1186/s40644-018-0178-0 (PMC6260899; doi:10.1186/s40644-018-0178-0)
Supplement: Supplementary file 3 — Table S1. Comparison of diffusion coefficients MD, Dmono, Dbi, Dtri. (PDF 682 kb) [file 40644_2018_178_MOESM3_ESM.pdf]

Table S1: Comparison of diffusion coefficients MD, D_{mono}, D_{bi}, D_{tri}

	**MD** [10^{-3} mm^2/s]	**D_{mono}** [10^{-3} mm^2/s]	**D_{bi}** [10^{-3} mm^2/s]	**D_{tri}** [10^{-3} mm^2/s]
**Healthy Cortex** (n=13)	2.16 (0.12)	2.15 (0.13)	1.93 (0.10)	1.41 (0.09)
**Healthy Medulla** (n=13)	2.21 (0.14)	2.24 (0.14)	2.02 (0.11)	1.55 (0.12)
**All solid lesions** (n=13)	1.94 (0.32)	2.03 (0.56)	1.71 (0.43)	1.39 (0.35)
**Cyst** (n=5)	3.04 (0.17)	2.95 (0.094)	2.90 (0.11)	2.74 (0.08)
**RCC** (n=11)	1.90 (0.32)	1.99 (0.57)	1.65 (0.40)	1.34 (0.33)
**cc-RCC** (n=9)	1.94 (0.33)	2.08 (0.60)	1.71 (0.42)	1.38 (0.34)
cc-RCC with sarcoid differentiation, extensive necrosis and hemorrhaging (n=1)	1.77	1.63	1.38	1.04
cc-RCC with extensive necrosis (n=1)	1.19	1.09	0.92	0.71
cc-RCC with papillary growth (n=1)	1.82	1.81	1.49	1.19
cc-RCC with cells situated in nests and extravasation of erythrocytes (n=1)	1.94	2.01	1.71	1.71
cc-RCC with areas of low cell density, hemorrhaging and cystic structures (n=1)	2.24	3.25	1.90	1.68
cc-RCC with extensive hemorrhage (n=1)	2.01	2.12	1.71	1.49
cc-RCC with cystic and solid areas (n=1)	2.20	2.17	1.97	1.69
cc-RCC with micro-cystic structures and hemorrhaging (n=1)	2.17	2.61	2.39	1.68
**p-RCC** (n=2)	1.68 (0.20)	1.57 (0.01)	1.36 (0.06)	1.14 (0.06)
Hemangioma (n=1)	2.38	2.62 (0.16)	2.45	1.99
Oncocytoma (n = 1)	1.98	2 (0.3)	1.63	1.36

RCC = renal cell carcinoma, cc-RCC = clear cell renal cell carcinoma, p-RCC = papillary renal cell carcinoma
